# Informed consent in dentistry and medicine in Spain: Practical considerations and legality

**DOI:** 10.4317/medoral.25265

**Published:** 2022-04-03

**Authors:** María Otero, Natsuki Oishi, Fernando Martínez, Maria Teresa Ballester, Jorge Basterra

**Affiliations:** 1 MD, PhD. Department of Anaesthesia, University General Hospital, Valencia, Spain; 2 MD, PhD. Department of otorhinolaryngology, University General Hospital, Valencia, Spain; 3 MD. Department of otorhinolaryngology, University General Hospital, Valencia, Spain; 4 MD, PhD. Surgery Department, Valencia Medical School, University of Valencia, Spain

## Abstract

**Background:**

The healthcare practice of dentistry, as well as medicine, is framed within a legal environment. Patients have the right to know all the information related to any action performed on them and dental or medical doctors are obliged to obtain their patient’s prior written informed consent (IC) before undertaking any healthcare procedures.

**Material and Methods:**

Here we reviewed the legality and jurisprudence in Spain regarding IC. We also used INFLESZ text readability analysis software to analyse a sample of official Spanish informed consent documents (ICDs) from different surgical and interventional procedures related to dentistry and oral cavity interventions.

**Results:**

It is a mistake to confound IC with ICDs. This error prevents physicians from considering the former as a care process in which the patient’s authorisation signature is the last link in a chain formed, almost in its entirety, by the informative process and deliberation alongside the patient. Multiple factors can influence communication between practitioners and their patients. Importantly, treatment adherence is greater when patients feel involved and autonomous in shared decision-making and when the circumstances of their lives are adequately considered. We concluded that although the ICDs we analysed conformed to the requirements set out in international law, they were somewhat difficult to read according to the reading habits of the general Spanish population.

**Conclusions:**

Knowledge about the legality of IC helps professionals to understand the problems that may arise from their non-compliance. This is because the omission or defective fulfilment of IC obligations is the origin of legal responsibility for medical practitioners. In this sense, to date, there have been more convictions for defective ICs than for malpractice. The information provided in ICs should include the risks, benefits, and treatment alternatives and must be tailored to the needs and capabilities of the patient to enable autonomous decision-making.

** Key words:**Informed consent, legality of informed consent, principle of autonomy, defensive medicine, satisfactive medicine, health law, stomatology, oral surgery, dentistry.

## Introduction

Obtaining informed consent (IC) is the end of a process of shared decision-making between health professionals and patients, in which the patient autonomously decides to take the course of action they consider to be best for them. This means that they must first receive all the appropriate information regarding their disease and all the diagnostic and/or therapeutic options available to them (1,2). The objective of this current manuscript was to take a practical approach to present a review of the theory, evolution, and Spanish legality of IC, with the aim of guiding dental or medical doctors in their daily clinical practice.

There are different types of IC (1-3). The most appropriate is the express form, which is manifested verbally, in writing, or by unequivocal signs. However, in many routine clinical acts, express consent is considered excessive, as occurs, for example, when doctors evaluate the results of an analysis or of a complementary test report. In these cases, tacit consent, understood by the professional based on the patient’s behaviour, is sufficient. However, tacit consent is not valid in cases where, by law, the patient’s will must be expressly indicated.

It is common to find patients who demand detailed information about their condition and treatments. The importance of IC is derived not only from its legal imperative. Patient satisfaction with the received information is a key indicator of effective communication, which itself drives patient behaviour (4). Providing information adapted to each case and its needs adjusts patient expectations by exposing them to the advantages and disadvantages of the proposed treatment. Indeed, a patient satisfied with the information provided to them is more likely to place greater trust in their doctor or dentist and become more involved in decision making. This correlates with a greater degree of patient involvement and adherence to treatment and therefore, with an improvement in therapeutic outcomes and prognosis (5-9). In fact, if a patient is not fully satisfied with the treatment administration mode or not convinced of its benefit, he will be less likely to comply with the prescribed regimen. Meanwhile, information is the best form of liability insurance for professionals because it helps protect them from lawsuits and convictions resulting from defective ICs (3,10-14).

## Material and Methods

We conducted a detailed analysis of Law 41/2002 on the basic regulation of patient autonomy and rights and obligations in terms of information and clinical documentation which represents the national standard that currently regulates the use of health information in Spain. The different regional rules on IC and jurisprudence on this subject, as well as some previous historical international documents and conventions (the Nuremberg Code, Declaration of Helsinki, Oviedo Convention, and Belmont Report) and their derivatives (Code of Ethics and Medical Deontology of the Collegiate Medical Organization of Spain) were also reviewed.

In addition, we undertook an index search in PubMed using the following search terms: [informed consent AND patient information AND informed consent legal AND health literacy]. Articles published from 2002 onwards (the date on which the revised national law was published) were included in this review. The collected information was analysed without the need for statistical tests and is presented here in the form of summaries and Tables (Table 1). Furthermore, we analysed a sample of official informed consent documents (ICDs) from different dental surgical and interventional procedures to verify their suitability from a legal standpoint and to check their readability by employing INFLESZ online software (https://legible.es/).

**Table 1:**
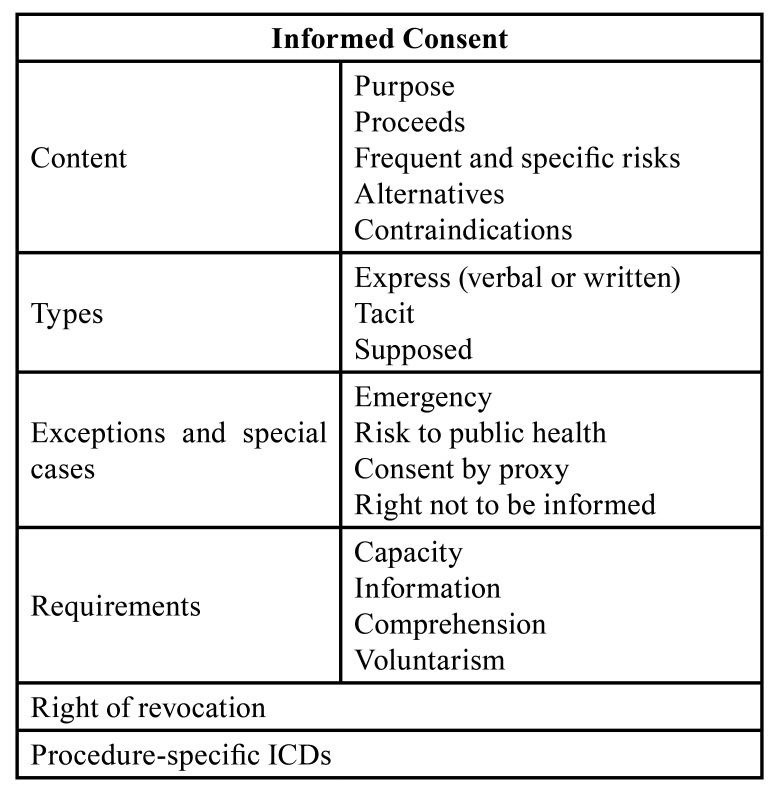
Relevant aspects of Informed Consent.

## Results

The topic of IC is one of the most studied and debated areas in the field of bioethics, with a huge number of books, scientific articles, and doctoral theses devoted to this subject (1,2,10,13-21). However, most of this work has focused on consent as a simple authorisation rather than on the ‘process’ of giving consent itself. The latter should be understood as the shared decision-making process between the two parties involved: the health professional and the patient (8,9,18). All the information is provided to the patient during the first part of this process while the patient deliberates and decides on their desired course of action in the second part. Here we reviewed the aspects of IC that mark Spanish Law and jurisprudence in this regard with the aim of summarising an approach useful both to doctors and dentists in their daily practice.

- Legislation

IC is extensively regulated at the international, national, and regional levels in many European countries (13,14). However, its legal roots reside in the Nuremberg Code from 1947 (22,23) after a group of doctors were accused of conducting experiments characterised as crimes against humanity on prisoners of war located in concentration camps during World War II. These experiments were carried out without providing information to the victims of the risks to which they were exposed or obtaining their prior consent. This was followed by the Declaration of Helsinki in 1964, the first Declaration of Patient Rights in 1973, the Oviedo Convention in 1977, and the Belmont Report in 1978. These are all considered historical milestones that established the ethical principles that guide doctor–patient relationships, as well as the patient’s right to receive complete information about their situation and treatment options. In Spain, this right was included in the first Code of Ethics and Medical Deontology from 1978 which was promulgated by the Collegiate Medical Organisation. Since then, this code has been updated three times, most recently in 2011, and is currently undergoing further revision.

Law 41/2002 on the basic regulation of patient autonomy and patient rights and obligations in terms of information and clinical documentation regulates, in a monographic manner, the autonomy of patients and IC in Spain, repealing the corresponding articles of General Health Law 14/1986. The following points are important aspects of Law 41/2002 which must be considered by dentists and doctors when undertaking their care activities.

a) Patients have the right to know all the available information about their health. This information should include the treatment risks, benefits, and alternatives, including the possibility of failure of the technique. Patients also have the right not to be informed, if that is their wish, but this desire must be expressed in writing. In this case, only the information necessary for the follow-up of the previously accepted prescribed treatment will be provided. However, this decision must be reported if it would put the patient or third parties at risk (such as in case of infectious-contagious diseases). The information provided to patients must be truthful, understandable, and appropriate to the needs and requirements of the patient, and will usually be provided verbally, leaving a written record in the clinical report.

b) Any intervention in the field of healthcare requires the free and voluntary consent of the affected person after having received the information and having evaluated their options. Even if the patient does not want to receive information, their prior consent must still be obtained before any procedure with the potential to affect their health. In the case of an emergency preventing IC from being obtained, a risk to public health, or therapeutic need, doctors may carry out essential clinical interventions in favour of the patient’s health in the absence of the IC. Subsequently, when circumstances permit, the patient or person responsible for their health must be informed and consulted. It is the responsibility of the professional to obtain consent, which may be verbal, except in the case of surgical acts or invasive procedures, where it must necessarily be in writing. Furthermore, the patient can revoke their consent at any time by leaving a written record of their decision to do so.

c) At the discretion of the doctor, consent by representation can be granted when the patient is unable to make decisions for themselves, when their decision-making capacity is judicially modified, when a patient is aged under 16 years and is not emancipated, or they are intellectually or emotionally unable to understand the scope of the intervention. In these cases, patients will be given information tailored to their degree of understanding and their opinion will be heard. If the patient lacks a legal representative, consent can be given by someone linked to them for family or factual reasons. The decision by representation must always consider the greatest benefit to the life or health of the patient in question. Any decisions that are contrary to these interests must be brought to the attention of the judicial authority. If this is not possible and the situation is urgent, health professionals will adopt the necessary measures to safeguard the life or health of the patient and will be protected by the causes of justification of compliance with their duty of care and the patient’s status of therapeutic need.

The legislation on this matter in the different Spanish regions is extremely varied, as also occurs in other European countries. The dental or medical doctor must combine both the basic regulations set by the state with the regional requirements in each case. This leads to conflicts when the regulations are not identical because some rules expand and develop aspects of national law while others do not. Thus, professionals who practice medicine or dentistry in different regions of the country must obtain different consents to perform the same interventions, depending on where they are providing care services.

A good example of this disparity is the contrast between Health Law 10/2014 of the Autonomous Community of Valencia and Law 3/2001 of the Autonomous Community of Galicia, which refers to the order of priority of individuals linked to the patient when granting consent by representation. Thus, the first law establishes the following order: (1) non-legally separated spouse or domestic partner; (2) the oldest of the next-degree relatives. However, if the patient had previously designated, in writing or in another undoubted way, a person for this purpose, the preference will correspond to that person. The second law also gives preference to the spouse or common-law partner and, failing that, to the closest-degree relatives, and adds that within the group of same-degree relatives, those who act as caregivers will have the preference, but makes no mention of the possibility of prior specific designations.

Another example in a different geographical location is as follows: Law 5/2010 of the Autonomous Community of Castilla-La Mancha indicates that, in serious cases involving patients who are minors, parents and/or their legal representatives will be informed, and their opinion will be considered in corresponding decision-making, even if the patient is aged over 16 years or is emancipated. In contrast, Law 21/2000 of the Autonomous Community of Catalonia only includes this obligation to report in cases of voluntary interruption of pregnancy, the practice of assisted human reproduction techniques, and in clinical trials, but not in all serious situations. However, Spanish national law indicates that the opinion of the parents or legal representatives must not only be considered but that they will also be asked to provide IC in serious cases, in addition to listening to the will of the minor. Furthermore, some regional laws extend this position: if the parents are separated or divorced, the IC must be provided jointly except in cases of vital emergency or everyday decisions that are not very important or are routine in the minor’s life and where the consent of the person present will suffice.

Although the national law 41/2002 does not determine when the care information must be provided or when the IC must be collected, it does indicate that it must be sufficiently in advance to guarantee the patient’s autonomous decision (14). However, Law 10/2014 of the Autonomous Community of Valencia expands that this information must be provided at least 24 hours before the corresponding procedure, except in urgent situations, and adds that in any case, the information cannot be given to the patient while they are numb, asleep, or their mental faculties are altered, nor when they are already inside the operating or procedure room where the intervention will be undertaken. Nonetheless, this clarification does not appear in other regional regulations. Therefore, IC and the signature of ICDs should be obtained sufficiently prior to the intervention to avoid obtaining them moments before the procedure, and in a suiTable place such as in general or consultation office areas.

- Jurisprudence

Some 90% of legal claims against doctors and dentists are related to defects in the transmission of information and in the IC. Analysis of the jurisprudence shows variability in their interpretations, and so it is difficult to determine the specific type and quantity of information professionals are obliged to give to patients so as not to incur malpractice complaints (3,11,12,24). Common law provides that health professionals must give only the information they believe appropriate rather than all the available material. However, this is not the trend in Spain. The content of the information that doctors or dentists must now provide for an IC to be considered correct is expanding under recent jurisprudence, even more in the case of so-called satisfactory medicine (25).

This is a type of medicine that does not treat pathologies and therefore, is not carried out on ill patients but rather, upon those who voluntarily desire interventions of various kinds to ‘improve’ their bodies. Dentistry has been conFigured mostly, by the courts, as a voluntary or satisfactory type of medicine. Examples of these voluntary procedures are teeth whitening or some types of orthodontics. In response to this trend in the courts, the recommended extent of the required IC is defined by the “necessity, severity, and novelty of medical interventions”. The less necessary an intervention is, the greater the amount of information that must be provided to the patient (3,25).

The omission or defective fulfilment of the IC generates civil liability giving rise to the possibility of compensation when the patient has experienced some degree of proven damage (1,3,12). Otherwise, this omission, or defective IC, would only constitute an infringement of professional duties with possible repercussions but without legal consequences. Therefore, damage is compensated, but in terms of the jurisprudence, there is no difference between the physical or moral damage caused by non-diligent provision of a medical intervention and the damage compensable for the absence of IC. We could say that the jurisprudence applies a ‘double standard’: when there is no physical damage, it argues that not all the risks and complications must be reported. However, when physical damage occurs and there has been no malpractice, it is argued the opposite, that exhaustive information must have been provided.

For the calculation of compensation, not only physical, but also moral damage is considered. Justice tries to compensate for disproportionate damages but, this same fact enhances defensive medicine. However, to understand that the right to information has been violated, the availability of a therapeutic alternative is not necessary. The damages derived from the lack of IC is also compensated in cases where there is a single treatment available for the disease. To claim otherwise would be to say that diseases that only have one treatment do not require IC. Consequently, cases where damage to the patient has occurred are also compensated, although it is considered proven that the person would have been operated or treated in the same way even if they had had all the relevant information.

The judicialisation of health has become a barrier to trust between patients and health professionals, thereby enhancing the importance of ICs, more as a tool to protect doctors from legal problems and claims than as a process in which decisions are made jointly and responsibly. The attempt by patients to obtain financial compensation for negative or unsatisfactory clinical results, sometimes with the support and encouragement of legal advisors, tends to result in the development of defensive practices by doctors and dentists because of the potential legal repercussions their work could have (11,26,27). All of this can lead to slower decision-making for fear of a subsequent legal claims.

- The informed consent documents

Although the informative function and formal requirements of ICDs are well known, in too many occasions their use still does not conform to current law. ICDs must be specific to each diagnostic or therapeutic procedure to be performed. Thus, generic ICDs are not adequate or legally accepted (28) and so, those whose headers do not correspond to the procedure to be performed are considered invalid in the eyes of the law. In addition to containing the information previously mentioned, they must also contain a section for the signature of the doctor or dentist, as proof that the verbal information was transmitted, and another for the signature of the patient or their legal representative, as proof of acceptance and consent. The date and a section for possible revocation of the IC must also be included, and finally, the patient must be given a copy.

The process of providing verbal information is a gradual process that can be carried out in one or more interviews and cannot be replaced by any written document (28-30). It is a mistake assume that IC is equivalent to ICD. This error prevents us from considering IC as a care process in which the patient’s authorizing signature represents the last link in a chain formed, almost in its entirety, by the informative process and deliberation of the patient. Another important issue is the readability of the ICDs professionals deliver to patients, which should be considered an indicator of quality of care. Readability is defined as the set of typographical and linguistic characteristics that allows readers to easily read and understand the information provided to them (22,31). Medical terminology often uses long words that are difficult to understand. Thus, to facilitate the understanding of health texts aimed at a general public with a variable level of health literacy, it is important to avoid the use of complex, extensive, and subordinate technical language and sentences in their elaboration (29).

In this work, we selected a sample of ICDs available from the different dental societies and verified that they were specific to each type of surgery and that they met the requirements set by law. In addition, their readability was analysed using INFLESZ online software (https://legible.es/). This program assigns the text a reading difficulty score from 0 to 100 using mathematical formulas to measure syntactic and semantic difficulty and to calculate the number of words, syllables, sentences, mean number of letters and syllables per word, average number of words per sentence, and the correlation with other readability indices.

The official interpretation of this index is as follows: 0–40, readability very difficult for an average Spanish citizen; 40–55, quite difficult; 55–65, normal; 65–80, quite easy; 80–100, very easy. The ICDs we analysed (for orthodontics, dental implantation, tooth filling, tooth extraction, endodontics, periapical surgery, pulp treatment in an immature tooth, periodontics, and oral surgery) had INFLESZ indices of 41.24 to 52.18 points, and so should all be considered somewhat difficult to read. In addition, they all scored below the cut-off point of 55 points, above which a text written in Spanish is considered to be accessible to the general public.

In summary, as part of the patient safety procedures, before initiating any surgical or interventional undertaking, patients or their legal representatives must provide a properly signed and verified ICD for the planned surgery or procedure and anaesthesia. Moreover, the doctors or dentists who will undertake the intervention must provide the information in the ICD sufficiently in advance of the procedure date, except in the case of an urgent intervention or if there is a potential risk to public health.

## Discussion

The patient’s right to information is independent of whether they decide to eventually undergo a health intervention. That is, the patient has the right to receive all the information about their health problem, regardless of whether a diagnostic-therapeutic decision has to be made at that time or not. This right to information precedes the IC (1,16), which can be considered the formal procedure for applying the principle of ‘autonomy’. Thus, the IC confers to the power to conduct procedures on a patient with their prior knowledge of the facts regarding the intervention and in the absence of coercion (3,23).

The information provided in ICDs should not only cover the risks inherent to each type of intervention, but also those related to the individual characteristics of each patient in relation to the age or the presence of other pathologies (2,10-14). This implies that when the patients undergo a procedure they already understand because they have previously consented and undergone the same intervention in the past (for example, a second dental implant), physicians are not exempt from the obligation to inform the patient and collect a new IC. This is because the patient’s specific risks may have changed and so they must be re-evaluated. Requests for additional information from the patient must be responded to appropriately. The patient must also be informed of their option to refuse treatment at any time, even after its initiation. Therefore, the IC process, which should be continuous and gradual, culminates with its acceptance or revocation, which will be accompanied by the signature of the ICD in the case of surgery or an invasive procedure (28-30).

Health professionals must be aware of the minimum requirements that, although not described by law, the IC must meet for it to be valid. These are: capacity, information, understanding, and voluntariness, or the absence of coercion (20,31-34). Capacity (competence in the legal field) is the individual’s ability to make decisions. However, making the distinction between competent and incompetent individuals can sometimes be complicated. Importantly, the way in which information is transmitted influences the way it is received and understood by patients, and this can condition any decisions derived from it (4,5). The understanding patients have of their disease or about a certain protocol depends a lot on its delivery mode and the words used, as well as the emphasis when this information is communicated (4,5).

The feeling of comfort and security derived from the professional’s capacity for empathy also plays a fundamental role in the IC process. This information must be sufficiently clear and formulated in a manner appropriate to the person undergoing the intervention so that they can adequately understand and weigh up the necessity or usefulness of the proposed procedure against the risks, burdens, or damage of any kind that it may entail. This implies that in the case of competent patients with learning disabilities, the information must be adapted, and relevant support measures must be provided—which could include adjusting the physical formats of the information—so that these individuals can exercise their right to autonomy.

Voluntariness refers to the fact that patients must freely decide to undergo treatment or participate in a study without being subjected to persuasion, manipulation, or coercion. The voluntary nature of consent is violated when it is requested by people in a position of authority or when the patient is not offered sufficient time to reflect, consult, or decide for themselves.

There are some exceptions to the need for IC, such as in situations of risk to public health or in an emergency which poses an immediate serious risk to the physical or mental integrity of the patient who is unable to express their will. In these cases, the need for IC may be ignored in order to perform an indispensable procedure that cannot be postponed. If the situation permits, the patient’s relatives or those de facto linked to them can be consulted, if they are present. There are also special cases such as consent by representation or substitution, where individuals linked to the patient for legal, family, or factual reasons can give IC when the patient is not able to do so due to physical or mental disability, legal disability, or because they are a minor. In the latter case, legally divorced parents who retain parental authority will both be represented, but in the case of routine medical decisions, the consent of the parent attending the medical centre with the minor will be sufficient. In the case of disagreement, a judge should be consulted.

Consent will be verbal in most situations, such as when blood pressure is taken or an electrocardiogram or oral cavity examination is performed. However, IC must be provided in writing in cases that involve noTable risks or inconveniences or that could have a foreseeable impact on the patient’s health such as surgical interventions or invasive diagnostic and therapeutic procedures. In these cases, the IC must be provided in a document on which the patient’s signature appears as an unequivocal sign of having received the verbal information directly from the specialist doctor. The more doubtful the outcome of an intervention or the less necessary it is for the patient’s health, the more important the patient’s prior written IC becomes (25).

The ICDs proposed by the different dental societies in Spain conform to law because they are specific to each procedure. However, these texts use complicated language that is difficult to read for the general Spanish population. Our analysis of the texts in a sample of these ICDs revealed that they were ‘somewhat difficult’ to read according to the INFLESZ index and therefore, they are not easily accessible to the entire population. Indeed, this finding is consistent with observations in other similar studies (30,33).

The fact that a patient signs an ICD does not mean that they have actually read it or understood the risks related to the proposed procedure (33,35). ICDs must transmit the information required to allow patients to participate in making decisions affecting their health (29), but these documents will only meet this objective if they are legible. The readability of ICDs can be improved by using shorter sentences and words, avoiding unnecessary medical technicalities, and by using subsections. The inclusion of graphics and explanatory drawings can also make it easier for the layperson to understand these documents.

The practice of oral medicine and oral surgery is framed within a legal environment in many countries. This review of the Spanish legality regarding this system clarifies the minimum content and other particularities of IC, which can vary in the different Spanish regions, thereby making this review useful for doctors and dentists alike (11-14,24). The judicialisation of health and reactive-defensive medicine are barriers to trust and good doctor–patient relationships (27). Patients must sign many consent documents before undergoing a medical procedure. Thus, the process of consenting can become excessively bureaucratised, sometimes leading it to become the mere act of signing a document written in difficult technical language that the patient, very often, did not even read.

This is a sign of defensive healthcare that seeks security against an increasing number of lawsuits against it. ICDs normalise and formalise relationships between healthcare professionals and patients. However, their use beyond the cases contemplated by law is related to the legal protection they provide to physicians. The information protects professionals from lawsuits and allows patients to be the protagonists of their own healthcare process by involving them in decisions. This also helps to improve the effectiveness of treatments by enhancing patient adherence to them (5-7). Importantly, the communicative skills of professionals plays a central role in the success of the IC process (4,5).
